# Inhibition of the Exocyst Complex Attenuates the LRRK2 Pathological Effects

**DOI:** 10.3390/ijms241612656

**Published:** 2023-08-10

**Authors:** Cristina Ciampelli, Grazia Galleri, Silvia Puggioni, Milena Fais, Lucia Iannotta, Manuela Galioto, Marta Becciu, Elisa Greggio, Roberto Bernardoni, Claudia Crosio, Ciro Iaccarino

**Affiliations:** 1 Department of Biomedical Sciences, University of Sassari, 07100 Sassari, Italy; c.ciampelli@phd.uniss.it (C.C.); galleri@uniss.it (G.G.); silvya97@tiscali.it (S.P.); faismilena@gmail.com (M.F.); galioto@uniss.it (M.G.); m.becciu1@studenti.uniss.it (M.B.); ccrosio@uniss.it (C.C.); 2 Department of Biology, University of Padova, 35131 Padova, Italy; lucia.iannotta@unipd.it (L.I.); elisa.greggio@unipd.it (E.G.); 3 Department of Pharmacy and Biotechnology (FABIT), University of Bologna, 40126 Bologna, Italy; roberto.bernardoni@unibo.it

**Keywords:** LRRK2, Sec8, exocyst complex, Parkinson’s disease

## Abstract

Pathological mutations in leucine-rich repeat kinase 2 (LRRK2) gene are the major genetic cause of Parkinson’s disease (PD). Multiple lines of evidence link LRRK2 to the control of vesicle dynamics through phosphorylation of a subset of RAB proteins. However, the molecular mechanisms underlying these processes are not fully elucidated. We have previously demonstrated that LRRK2 increases the exocyst complex assembly by Sec8 interaction, one of the eight members of the exocyst complex, and that Sec8 over-expression mitigates the LRRK2 pathological effect in PC12 cells. Here, we extend this analysis using LRRK2 drosophila models and show that the LRRK2-dependent exocyst complex assembly increase is downstream of RAB phosphorylation. Moreover, exocyst complex inhibition rescues mutant LRRK2 pathogenic phenotype in cellular and drosophila models. Finally, prolonged exocyst inhibition leads to a significant reduction in the LRRK2 protein level, overall supporting the role of the exocyst complex in the LRRK2 pathway. Taken together, our study suggests that modulation of the exocyst complex may represent a novel therapeutic target for PD.

## 1. Introduction

Mutations in leucine-rich repeat kinase 2 gene (LRRK2, PARK8) are the most frequent genetic cause of Parkinson’s disease (PD), reaching up to 40% in some ethnic groups [1]. The incomplete age-dependent penetrance [2] underlies the complex interplay between genetics, environment, and age in PD development. LRRK2 is a large multidomain protein belonging to the ROCO family protein characterised by a Ras-like GTPase domain, called Roc, followed by a COR (C-terminal of Roc) domain. LRRK2 contains different protein interaction modules: armadillo repeats (ARM), ankyrin repeats (ANK), leucine-rich repeats (LRR), and WD40 domain [3]. The LRRK2 pathological mutations are autosomal dominant and clustered around the central catalytic core (Roc, COR, or kinase domains) of the protein. Moreover, two LRRK2 mutations that act as PD risk factors have been identified: one in the COR domain and one in the WD40 repeats [3]. Clinically, PD patients carrying LRRK2 mutations are often indistinguishable from idiopathic ones, although some differences are observed at a neuropathological level, with a prevalence of Tau aggregates in LRRK2 mutation carriers [4].

Currently, the LRRK2 physiological and pathological function remains enigmatic despite the development of many cellular and animal experimental models based on LRRK2 over-expression, knock-in, or knock-out. Although the LRRK2 function has been linked to different cellular pathways, there is a large consensus on a key role of LRRK2 in controlling vesicle trafficking [5]. Interestingly, an alteration in synaptic vesicle trafficking could be a common pathological mechanism in PD [6,7]. A subset of RAB GTPase, including RAB3, RAB8, RAB10, and RAB35, is a substrate of LRRK2 kinase activity [8]. RABs are well-known regulators of intercompartmental transport of vesicles carrying cargos in and out of cells and organelles [9]. Accordingly, RABs modulate different neuronal structures/functions, ranging from the addition of membrane and proteins during neurite outgrowth or mature synapses, the formation of the correct neuronal contacts during the development to different key functions in differentiated neurons including protein or neurotransmitter secretion, and organelle biogenesis. In addition to RABs, LRRK2 has been shown to interact and, in some cases, phosphorylate other proteins involved in vesicle dynamics, including DNAJC6 (auxilin) [10], synaptojanin1 [11], NSF [12], and EndoA [13].

Different pathological phenotypes associated with mutant LRRK2 expression may be explained by an alteration in vesicle trafficking. For instance, we have previously demonstrated that LRRK2 modulates dopamine receptor trafficking [14]. LRRK2 regulates dopamine levels, presynaptic glutamate release via Dopamine Receptor D2 (DRD2)-dependent synaptic plasticity, and dopamine-receptor signal transduction [15]. Matikainen-Ankney et al. highlighted a fourfold increase in spontaneous excitatory postsynaptic currents (sEPSC) frequency in the dorsal striatal spiny projection neurons leading to an alteration of postsynaptic structures into striatum in mice expressing LRRK2 G2019S knock-in (KI) mutations [16]. In addition, LRRK2 G2019S KI mice displayed elevated glutamate and dopamine transmission and aberrant D2-receptor responses, although the number of synapses or spine-like structures were not altered [17]. Interestingly, mice expressing the LRRK2 G2019S mutant, inducible by tetracycline, showed a significant reduction in synaptic vesicle number and a greater abundance of clathrin-coated vesicles in DA neurons [18].

Recently, LRRK2 has been implicated in cilia formation. In LRRK2 R1441C mice, cholinergic interneurons of the dorsal striatum show a significant deficit in cilia formation through a reduction in glial-derived neurotrophic factor (GDNF) secretion triggered by Sonic Hedgehog (SHH) secreted by dopaminergic neurons for a reciprocal neuroprotection activity [19]. Interestingly, different genes are involved in both vesicle trafficking and autophagy, strongly indicating that the modulation of vesicle dynamics is a critical factor also in autophagic processes [7]. Among the protein actors modulating vesicle trafficking, a key function is played by the exocyst complex, an evolutionarily highly conserved complex composed of eight different members (Sec3, Sec5, Sec6, Sec8, Sec10, Sec15, Exo70, and Exo84 [20]). The exocyst complex is involved in a variety of biological pathways, including tethering of secretory vesicles, derived from the trans-Golgi network (TGN) or recycling endosomes (RE), to the plasma membrane upstream of the SNARE proteins. For instance, different members of the exocyst complex interact with SNARE members or SNARE-interacting proteins [21], and this interaction precedes the vesicle docking to the receiving membrane before vesicle lipid fusion. In cultured hippocampal neurons, the exocyst complex is present in regions of ongoing membrane addition: the tips of growing neurites, filopodia, and growth cones [22]. In fact, the exocyst complex seems to play a prominent role in neuronal cell polarity, since the absence of functional exocyst subunits significantly alters neurite outgrowth in different biological systems [23,24].

In this context, we have previously demonstrated that LRRK2 interacts with Sec8 and positively modulates the exocyst complex formation [25]. This effect is due to its kinase activity, since it is ablated by specific LRRK2 kinase inhibitors or by LRRK2 kinase-deficient mutants [25]. Interestingly, exocyst and RABs (including LRRK2 targets RAB8 and RAB10) are part of the same protein complex that couples the generation of secretory vesicles at donor compartments to their docking and fusion [26]. The exocyst complex is regulated by small GTPases of the Rho, Ral, and Rab families [27]. While Rho and Ral GTPases mainly regulate the exocyst function on target membranes, the Rab family of small GTPases contributes to vesicle dynamics by regulating the assembly of the exocyst complex on vesicles prior to their arrival to target membranes. For instance, Sec4 and its mammalian homolog Rab8, which are important Rab GTPases present on secretory or recycling vesicles, directly interact with the exocyst member Sec15 [28]. Notably, the exocyst complex is required for some, but not all, forms of exocytosis; for instance, mutations in the exocyst component Sec5 impair the cell growth and membrane protein insertion without significant alteration in neurotransmitter release [29].

The exocyst complex function is highly conserved in all eukaryotes including drosophila. For instance, in this experimental system, apical secretion of Wnt1, a glycosylated protein secreted from various cell types, is inhibited by knockdown of Sec6 and Sec8 in polarised epithelial [30]. Moreover, the exocyst complex is essential in apical exocytosis of epithelial photoreceptor cells [31], or for the proper vesicular trafficking and membrane addition in anaphase cell elongation and cytokinesis [32]. Interestingly, in drosophila, a partial loss of Sec5 function partially suppresses the toxicity poly(GR) [33], a toxic dipeptide repeat protein produced from the GGGGCC repeat expansion in the C9ORF72 gene, which is responsible for amyotrophic lateral sclerosis and frontotemporal dementia.

Sec8 gene ablation is lethal in both mice and drosophila. In mice, it is early embryonic lethal since mutant embryos initiate gastrulation but are unable to progress beyond the primitive streak stage [24]. In drosophila, the lethality is also associated with a significant defect in the development of glutamatergic neuromuscular junctions (NMJs) [34]. Interestingly, in primary mouse neurons, FGF receptor-mediated phosphorylation of Sec8 is required for the efficient recruitment of the exocyst complex and regulates the addition of the new membrane to the cell surface of growth cones in developing neurons [35]. Moreover, Sec8 modulates NMDA receptor trafficking by its interaction with synapse-associated protein 102 (SAP102) [36].

In general, the asymmetric distribution of proteins and lipids is essential for a variety of cellular functions. In neurons., this aspect is still more relevant due to the elaborate neuronal structure, with axon and dendritic terminals branching out to great distances from the cell body, and local recycling of membrane and proteins has a tremendous impact in maintaining the fast kinetics of neuronal signal transmission. In addition, the exocyst complex is present at multiple subcellular locations and has been implicated in the regulation of other biological processes that could be important for neuronal physiology: endocytosis, autophagosome biogenesis, phagocytosis, and DNA repair [21].

In the present study, we show that the modulation of the exocyst complex by LRRK2 is likely mediated by RAB phosphorylation. Moreover, the treatment with Endosidin2, an exocyst complex inhibitor, rescues the LRRK2 pathological phenotype both in cells and in drosophila LRRK2 models. Importantly, prolonged Endosidin2 treatment leads to a reduction in the LRRK2 protein level, suggesting a mutual interaction between LRRK2 and the exocyst complex.

## 2. Results

### 2.1. LRRK2 Effect on Exocyst Complex Association Is Likely Dependent on RAB Phosphorylation

**Figure 1:**
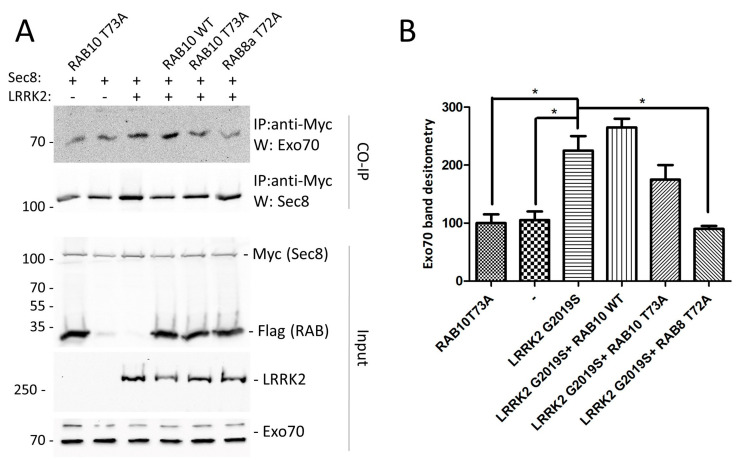
Evaluation of the effect of RAB8 or RAB10 phospho-deficient mutant expression on exocyst complex assembly mediated by LRRK2. (**A**) Analysis of mutant RAB expression on Sec8–Exo70 association. HEK293 cells were transfected with Myc–Sec8 in the presence or not of the indicated plasmids. Forty-eight hours later, cells were lysed and the protein extracts were subjected to a co-immunoprecipitation experiment using an anti-Myc antibody. Immunoprecipitated proteins were visualised by Western blot using an anti-exo70 antibody. The membrane was then incubated with an anti-Flag antibody to assess the efficiency of the immunoprecipitation. Finally, protein extracts were used to visualise Sec8, RABs (anti-Flag), LRRK2 (anti-LRRK2), or exo70. (**B**) Relative band densitometry for Exo70 of data obtained in (**A**) normalised to cells transfected by Sec8 alone. The data represent the mean ± SEM. * *p* < 0.05. One-way ANOVA followed by Bonferroni post-test was used.

We have previously demonstrated that LRRK2 over-expression mediates exocyst assembly and this effect is dependent on LRRK2 kinase activity [25]. In an attempt to clarify whether the modulation of the exocyst complex by LRRK2 is dependent on RAB phosphorylation, we evaluated the Sec8–Exo70 association in the presence of LRRK2 and phosphor-deficient RAB8 (RAB8T72A) or RAB10 (RAB10T73A) by a co-immunoprecipitation (co-IP) experiment. Specifically, HEK293 cells were co-transfected with LRRK2 and Myc–Sec8 in the presence or absence of RAB8T72A or RAB10T73A and the association between Sec8 and Exo70 was evaluated via co-IP and Western blot with an anti-Exo70 antibody. As illustrated in Figure 1A,B, we confirmed the increase in the Sec8–Exo70 protein association in the presence of LRRK2, while the expression of RAB8T72A and to a lesser extent, even if not significantly, RAB10T73A, significantly reduced the LRRK2 effect, suggesting that the modulation of the exocyst complex assembly by LRRK2 is downstream of LRRK2-mediated RAB phosphorylation.

### 2.2. Effect of Sec8 Over-Expression in LRRK2 Drosophila Models

**Figure 2:**
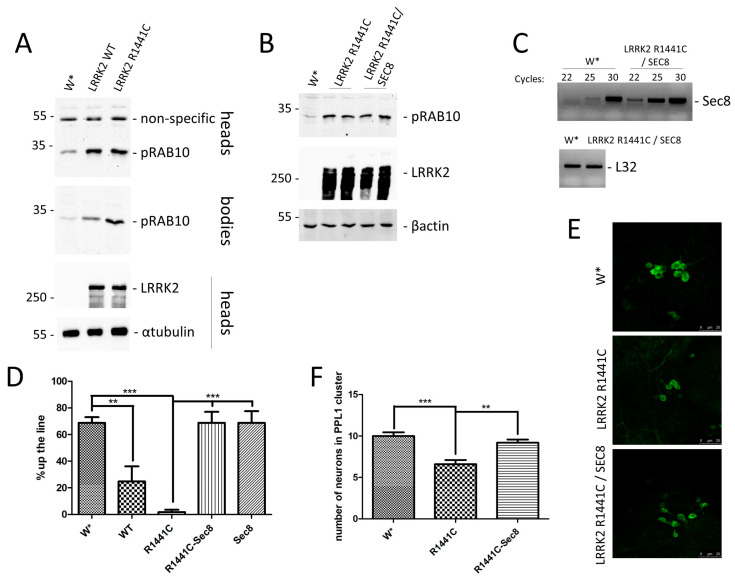
Evaluation of Sec8 over-expression in LRRK2 drosophila models. (**A**) Characterisation of drosophila lines expressing LRRK2 WT or R1441C under the control of Actin-GAL4 driver. One-week-old flies with the indicated genotypes were sacrificed and heads and bodies were dissected. Protein extracts were separated by SDS-PAGE and analysed by Western blot using anti-pRAB10, anti-LRRK2, and α-tubulin. (**B**) Characterisation of drosophila lines expressing LRRK2 R1441C or LRRK2 R1441C/dSec8 under the control of Actin-GAL4 driver. One-week-old drosophila were sacrificed and analysed as previously described. (**C**) Analysis by RT-PCR of dSec8 over-expression in LRRK2 R1441C/dSec8 under the control of Actin-GAL4 driver. PCR samples at 22, 25, and 30 cycles (for dSec8) and 22 cycles (for ribosomal protein L32) analysed on agarose gels are shown. (**D**) Evaluation by climbing assay of locomotor activity of 45-day-old drosophila lines expressing LRRK2 R1441C, LRRK2 R1441C/dSec8, dSec8, or W* under the control of Actin-GAL4 driver. The data represent the percentage of drosophila reaching 8 cm in 10 s from three independent experiments and are represented as mean ± SEM. At least 20 drosophila have been analysed for each biological replicate. ** *p* < 0.01; *** *p* < 0.001. One-way ANOVA followed by Bonferroni post-test was used. (**E**) Dopaminergic staining of PPL1 area of 45-day-old drosophila lines expressing LRRK2 R1441C, LRRK2 R1441C/dSec8, or W^+^ by immunofluorescence on whole brains using anti-TH antibody. (**F**) Quantification of dopaminergic neuronal number in PPL1 cluster of the different genotypes. The data represent the numbers of dopaminergic cells of three independent experiments and are represented as mean ± SEM. At least five brains have been analysed for each biological replicate. ** *p* < 0.01; *** *p* < 0.001. One-way ANOVA followed by Bonferroni post-test was used.

Since different LRRK2 drosophila models have been generated [37], we evaluated climbing defects in flies expressing mutant LRRK2 under a neuronal driver (Elav- or nSYB-GAL4) or under a constitutive driver (Actin- or Tubulin-GAL4). As shown in Supplementary Figure S1, we were able to detect a significant effect of LRRK2 mutant expression starting from 30 days post eclosion (Figure S1B), with only the two different drivers expressing GAL4 under a constitutive promoter. Thus, we used the Actin-GAL4 driver for all subsequent experiments. Bioinformatic analysis revealed identical aminoacidic sequence around the threonine 73 of RAB10 between human and drosophila sequence and a previous research study confirmed the ability of human LRRK2 to phosphorylate the drosophila homologue of RAB10 [38]. Thus, we evaluated the increase of endogenous dRAB10 phosphorylation in our drosophila lines. As shown in Figure 2A, both LRRK2 WT and R1441C expression results in a significant increase in dRAB10 phosphorylation in drosophila heads and whole bodies, strongly supporting this drosophila model for the study of the LRRK2 physio-pathological function.

The LRRK2 R1441C mutant shows a higher RAB10 phosphorylation level compared to LRRK2 WT in whole bodies, while no significant differences were detected using head protein extracts. This last result may be explained by the highest level of RAB10 basal phosphorylation in the heads compared to the whole bodies. One possibility is that RAB10s phosphorylation is already saturated in the presence of LRRK2 WT and no further phosphorylation may be achieved by the R1441C mutant. Interestingly, this experimental model also shows a significant reduction in locomotor activity measured by climbing assay starting from 30 days, while no differences were detected at 7 days (Figure S1A,B).

To analyse the Sec8 over-expression effect on LRRK2 drosophila models, we generated a drosophila line expressing both the LRRK2 R1441C mutant and dSec8. As illustrated in Figure 2B,C, Actin-GAL4 can drive the expression of both LRRK2 (detected by Western blot) (Figure 2A) and Sec8 (detected by RT-PCR) (Figure 2C), and the dSec8 over-expression does not affect either LRRK2 expression (Figure 2C) or dRAB10 phosphorylation (Figure 2C), further supporting that the exocyst complex is downstream of the RAB signalling pathway.

At 45 days, the LRRK2 R1441C transgenic line shows a significant reduction in locomotor activity compared to controls measured by the climbing assay (Figure 2D). Importantly, the concomitant expression of Sec8 significantly rescues the LRRK2 R1441C locomotor phenotype (Figure 2D). The analysis of the dopaminergic neuron cluster in the posterior inferiorlateral proto-cerebrum (PPL1) of adult brains from these drosophila lines reveals a significant reduction of dopaminergic neurons in LRRK2 R1441C expressing lines compared to controls. Furthermore, the concomitant over-expression of Sec8 also significantly rescues this phenotype (Figure 2E,F).

### 2.3. Effect of Endosidin2 on LRRK2 Pathological Phenotype in Both Cell and Animal Models

**Figure 3:**
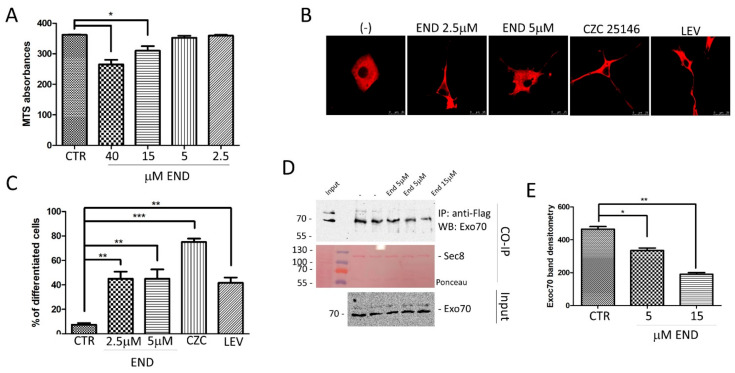
Analysis of Endosidin2 treatment on neurite branching in PC12 cells expressing LRRK2 G2019S. (**A**) Analysis of Endosidin2 toxicity by MTS assay in PC12 cells. The cells were treated with Endosidin2 at indicated concentrations for 48 h, then MTS compound was added and the absorbance was measured by a multi-plate reader. The data, from n = 3 independent cultures, are presented as mean ± SEM. * *p* < 0.05. One-way ANOVA followed by Bonferroni post-test was used. (**B**) PC12 cells stably expressing dox-inducible LRRK2 G2019S were treated for 7 days with NGF and doxycycline in the absence or presence of two different concentrations of Endosidin2. The LRRK2 kinase inhibitor (CZC 25146) and SV2A binding compound (Levetiracetam) were used as positive controls. (**C**) Quantification of data obtained in (**B**). The data represent the numbers of cells showing evident neurite extensions from three independent experiments and are represented as mean ± SEM. At least 20 cells have been analysed for each biological replicate ** *p* < 0.01; *** *p* < 0.001. One-way ANOVA followed by Bonferroni post-test was used. (**D**) Effect of Endosidin2 treatment on exocyst complex association analysed in HEK293 cells transfected by Flag-Sec8 WT. After transfection, the cells were treated for 24 h by Endosidin2 at the indicated concentrations, then the protein extracts were used for a co-IP experiment using an anti-Flag antibody to isolate Sec8. The co-immunoprecipitated proteins were visualised by Western blot using an anti-Exo70 antibody. Membranes were stained with Red Ponceau to confirm Sec8 immunoprecipitation. The input fraction was incubated by anti-Exo70 as control. (**E**) Relative band densitometry for Exo70 of data obtained in (**D**) normalised to cells transfected by Sec8 alone. The data represent the mean ± SEM. * *p* < 0.05; ** *p* < 0.01. One-way ANOVA followed by Bonferroni post-test was used.

We have previously demonstrated that an increase of the exocyst complex assembly is stimulated by LRRK2 over-expression [25] and, importantly, this effect is mediated by LRRK2 kinase activity [25]. Recently, Endosidin2, a small molecule compound able to impair the exocyst complex, has been developed [39]. Endosidin2 binds to Exo70, inhibiting the exocyst function in exocytosis and resulting in cell toxicity at high doses [39,40]. To evaluate if Endosidin2 has any effect on LRRK2 phenotype, we first assessed Endosidin2 cell toxicity in PC12 cells by MTS assay. At 40 and 15 µM, the compound is significantly toxic, while no differences in cell viability were observed at 5 and 2.5 µM after 48 h of treatment (Figure 3A) compared to untreated cells.

Prompted by the previous results, we analysed the effect of 5 or 2.5 µM Endosidin2 on LRRK2 phenotype using, as experimental assay, the well-established inhibitory effect of LRRK2 G2019S on neuronal differentiation [41]. First, in a series of preliminary experiments, we treated PC12 cells with Endosidin2 (5 or 2.5 µM) and no significant effect on NGF-induced differentiation was detected. PC12 cells expressing doxycycline-inducible LRRK2 G2019S [41] were then treated for 7 days by NGF in the presence or absence of Endosidin2. LRRK2 kinase inhibitor (CZC-25146) and Levetiracetam [41] treatments were used as a positive control. Cells were fixed, stained with anti-LRRK2 antibodies, and neurite outgrowth was analysed by confocal microscopy. Interestingly, as with LRRK2 kinase inhibition, treatment with Endosidin2 rescued the inhibitory effect of LRRK2 G2019S on neurite outgrowth (Figure 3B,C). To be sure that low doses of Endosidin2 may still influence the exocyst complex assembly, we performed a co-immunoprecipitation experiment. HEK293 cells were transfected by Flag-Sec8 and treated or not with Endosidin2 for 24 h at the indicated concentrations. Protein extracts were used for immunoprecipitation with anti-Flag to pull-down Sec8 and the associated Exo70 was visualised by Western blot. As illustrated in Figure 3D,E, in the presence of Endosidin2, a significant reduction in the Sec8–Exo70 complex formation is detectable already at 5 µM.

Finally, we extended our analysis to the previously described in vivo LRRK2 drosophila models, to evaluate the Endosidin2 effect on both locomotor activity and dopaminergic neuron number. In a preliminary experiment, we evaluated the effect of 50 or 150 µM Endosdin2 on fly vitality and climbing at 7 and 45 days after eclosion. No significant effect was detected in the presence of both Endosidin2 doses, so we decided to use the highest dose for the treatment LRRK2 R1441C transgenic line.

**Figure 4:**
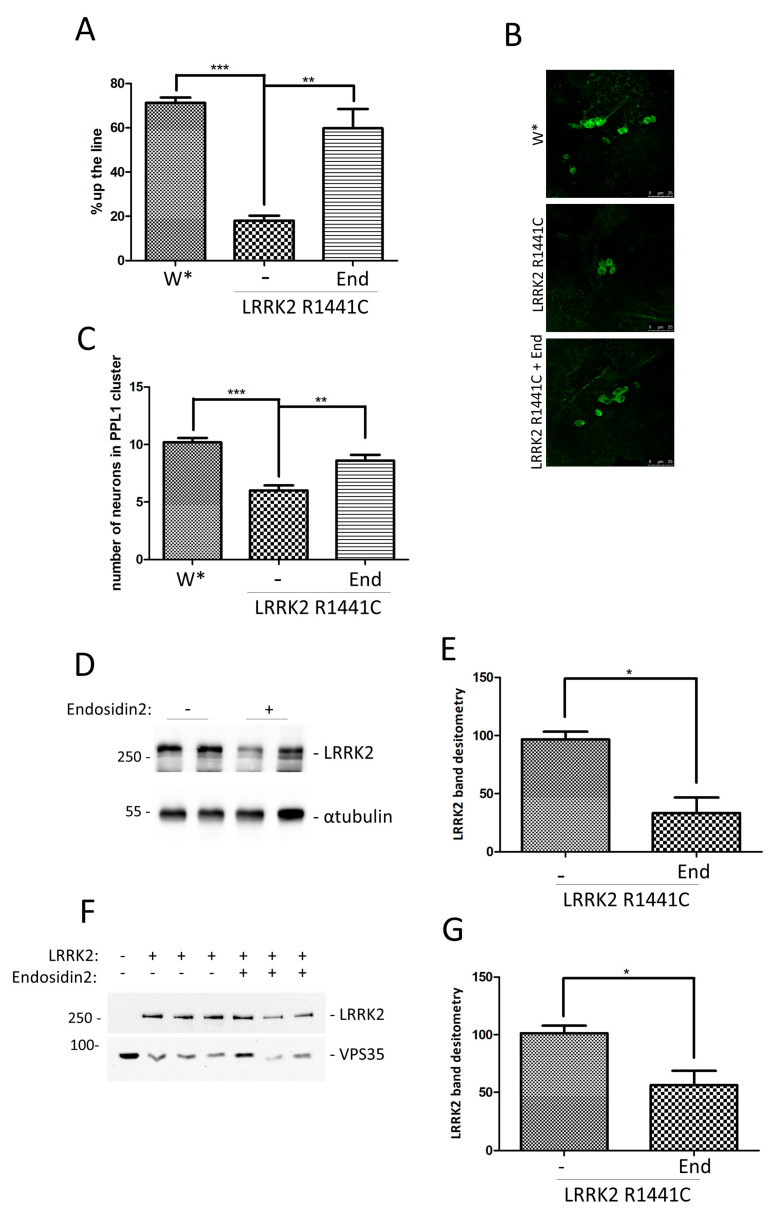
Analysis of Endosidin2 treatment on LRRK2 drosophila models and Edosidin2 effect on LRRK2 protein levels. (**A**) Evaluation by climbing assay of locomotor activity of 45-day-old drosophila lines W^+^ or LRRK2 R1441C crossed with Actin-GAL4 drivers treated or not by Endosidin2. The data represent the percentage of drosophila reaching 8 cm in 10 s of three independent experiments and are represented as mean ± SEM. At least 20 drosophila were analysed for each biological replicate. ** *p* < 0.01, *** *p* < 0.001. One-way ANOVA followed by Bonferroni post-test was used. (**B**) TH staining of PPL1 area of 45-day-old drosophila lines expressing LRRK2 R1441C under the control of Actin-GAL4 treated or not with Endosidin2 by immunofluorescence on whole brains using anti-TH antibody. W* was used as control. (**C**) Quantification of dopaminergic neuron number in PPL1 cluster of the different genotypes. The data represent the numbers of dopaminergic cells of three independent experiments and are represented as mean ± SEM. At least five brains have been analysed for each biological replicate. ** *p* < 0.01; *** *p* < 0.001. One-way ANOVA followed by Bonferroni post-test was used. (**D**) Analysis by Western blot of Endosidin2 effect on LRRK2 protein level, in LRRK2 drosophila models. The Actin-GAL4/R1441C flies were treated with Endosidin2 (150 µM) for 5 days, then protein extracts were prepared for Western blot analysis using an anti-LRRK2 antibody. Anti-tubulin was used as loading control. (**E**) Relative band densitometry for LRRK2 of data obtained in (**E**) normalised to tubulin. LRRK2 level in untreated flies is indicated as 100%. The data represent the mean ± SEM. * *p* < 0.05. The Student’s *t*-test was used. (**F**) Analysis of Endosidin2 effect by Western blot on LRRK2 protein level in SH-SY5Y cells. Cells, in duplicates, were transduced with LRRK2 recombinant adenovirus and treated with Endosidin2 (5 µM) for 72 h, then protein extracts were prepared for Western blot analysis using an anti-LRRK2 antibody. Anti VPS35 was used as loading control. (**G**) Relative band densitometry for LRRK2 of data obtained in (**F**) normalised to VPS35. The data represent the mean ± SEM. * *p* < 0.05. The Student’s *t*-test was used.

We treated or not Actin-GAL4/white or Actin-GAL4/LRRK2 R1441C flies with Endosidin2 (150 µM), starting 3 days after eclosion. At 45 days of age, the different drosophila lines were evaluated for climbing activity and dopaminergic neuron number. We confirmed the significant reduction in locomotor activity in drosophila expressing LRRK2 R1441C and we demonstrated that chronic Endosidin2 treatment significantly rescues the climbing defects in R1441C transgenic flies (Figure 4A).

Moreover, chronic Endosidin2 treatment also rescued the decrease in dopaminergic neurons in drosophila LRRK2 brains (Figure 4B,C).

### 2.4. Prolonged Endosidin2 Treatment Reduces the LRRK2 Protein Level

To further investigate the molecular mechanism by which Endosidin2 may act on the LRRK2 pathway, we started analysing the LRRK2 protein level upon Endosidin2 treatment, both in drosophila lines and SH-SY5Y neuronal cells. The R1441C LRRK2/Actin-GAL4 flies were fed for 5 days with 150 µM Endosidin2 and then sacrificed. The LRRK2 protein level was evaluated by Western blot. Surprisingly, as illustrated in Figure 4D,E, the Endosidin2 treatment determines a significant reduction in the LRRK2 protein level. We performed a similar experiment in SH-SY5Y neuronal cells, where LRRK2 expression was driven by a low titer adenovirus recombinant system to obtain a more physiological expression compared to transfection expression systems. SH-SY5Y cells were transduced and 24 h later treated by Endosidin2 for a further 72 h in 1% serum. The cells were lysed and the LRRK2 level was evaluated by Western blot. As illustrated in Figure 4F,G, treatment by 5 µM Endosidin2 determines a significant reduction in the LRRK2 protein level. To clarify whether the Endosidin effect was due to a rapid increase in the LRRK2 degradation rate, we performed a similar experiment in which protein translation was blocked by puromycin treatment. After transduction with LRRK2, cells were treated with Endosidin2 in the presence or absence of puromycin for 4 or 8 h. In the presence of Endosidin2, the half-life of the LRRK2 protein is not significantly affected (Figure S1C). The result suggests that the reduction in the LRRK2 protein level after prolonged exposure to Endosidin2 may be a long-term effect of inhibition of the exocyst complex.

## 3. Discussion

Experimental evidence, in different LRRK2 cellular and animal models, highlights a prominent role of LRRK2 in the control of vesicle trafficking [5,42] at multiple levels including the exocyst complex formation [25]. The exocyst complex is a key component of exocytosis processes regulating the vesicle trafficking from Golgi to cell membrane. In neurons, this pathway precedes the SNARE complex formation and, indeed, several members of the exocyst complex can interact with SNARE members or SNARE-interacting proteins. Thus, since vesicle binding to exocyst precedes fusion, temporal and spatial control of exocytosis in cells can be accomplished through exocyst regulation. Moreover, the exocyst complex has been implicated in the regulation of other biological processes important for neuronal physiology: endocytosis, autophagosome biogenesis, phagocytosis, and DNA repair [21].

Importantly, exocyst subunits have been found to be direct targets of a number of GTPases, including RAB10 e RAB8 [26,43,44]. In this research paper, we demonstrated that the effect of LRRK2 on the exocyst complex is likely downstream of LRRK2-mediated RAB phosphorylation. In fact, phospho-deficient RAB8 and partially, although not significantly, phospho-deficient RAB10 over-expression reduces the LRRK2 effect on exocyst assembly (Figure 1). Although both RAB8A and RAB10 are localised in the tubular perinuclear endocytic recycling compartments and regulate a variety of membrane trafficking events including exocytic polarised targeting and endocytic recycling events, they have a partial but not redundant function [9].

Interestingly, we were able to confirm in vivo, using a drosophila model, that Sec8 over-expression reduces the LRRK2 pathological phenotype, as previously demonstrated in neuronal PC12 cells [25]. First, we showed that the LRRK2 expression under the control of the Actin-GAL4 driver determines a significant increase in endogenous dRAB10 phosphorylation, suggesting the activation of similar biological pathways compared to eukaryotic models. Finally, we demonstrated that Sec8 over-expression may significantly rescue the LRRK2 R1441C pathological phenotype, both in terms of motor activity (climbing assay) and dopaminergic neuron numbers. The molecular mechanism by which Sec8 over-expression counteracts the LRRK2 pathological phenotype is quite cryptic and far from obvious. We favour the hypothesis that over-expressed Sec8 may interact with LRRK2 impairing its interaction and, consequently, the modulation of the endogenous exocyst complex, although different and alternative hypotheses can be formulated. For instance, the over-expression of a specific member may alter the overall formation of the exocyst complex by sequestering some specific components.

Interestingly, a mild inhibition of the exocyst complex, by Endosidin2 treatment, significantly rescues the LRRK2 pathological phenotype in both PC12 cells and drosophila models. Endosidin2 specifically targets the conserved exocyst complex subunit Exo70 to inhibit exocytosis [39]. In several experimental models, Endosidin2 is toxic, probably due to a complete block of exocyst complex-mediated vesicle secretion. In both PC12 cells and drosophila models, we specifically identified an Endosidin2 dose that does not affect cell or fly viability, but still significantly affects the exocyst complex assembly. In a PC12 differentiation assay, a low dose of Endosidin2 significantly reduces the LRRK2 G2019S inhibitory effect on PC12 neurite outgrowth (Figure 3). Similar results were obtained in LRRK2 R1441C drosophila models where chronic Endosidin2 treatment significantly rescued both climbing activity and dopaminergic neuron number analysed in 45-day-old flies (Figure 4A–C). Interestingly, Endosidin2 treatment has a long-term effect in reducing the LRRK2 protein level in both drosophila and SH-SY5Y neuronal cells (Figure 4D–G), strongly suggesting a mutual interaction between LRRK2 and the exocyst complex. LRRK2 and the exocyst seem to be part of the same protein complex, where LRRK2 modulates the exocyst complex formation likely by RAB phosphorylation and a prolonged inhibition of the exocyst complex assembly leads to a decrease in the LRRK2 protein level.

The complex processes of intracellular trafficking are carefully orchestrated by a plethora of protein and non-protein components, and the chemical manipulation of the trafficking machinery is of particular interest in many different biomedical areas. For instance, there is mounting evidence that vesicle trafficking, including the release of extracellular microvesicles, is a highly important biological process and could be a potential target for therapeutic intervention in tumorigenesis [45] and immune-mediated diseases [46].

To date, the LRRK2 effect in increasing or decreasing vesicle dynamics is still debated and highly dependent on the experimental model. Taken together, all our data strongly suggest that a mild inhibition of vesicle trafficking may alleviate the LRRK2 pathological effect in both neuronal cell lines and drosophila models. Targeting the exocyst complex or, more generally, proteins involved in vesicle dynamics could be a possible therapeutic option for PD treatment. For instance, recent in vitro studies support the idea that cell-to-cell propagation of α-syn significantly contributes to pathological changes in synucleinopathies [47]. Preventing the early events of transcellular spread, including exosomes, of α-syn across membranes and/or α-syn uptake may be a novel approach to halt disease spreading in PD and other synucleinopathies. However, blocking α-syn uptake/endocytosis could be the best therapeutic option since accumulating experimental evidence indicates that exosomal secretion of α-synuclein may be a protective mechanism to eliminate intracellular α-syn in parallel with autophagic degradation [48]. Unfortunately, the inventory of small molecules that affect vesicle trafficking is rather limited [49], but it may be worthwhile to screen for new molecules and to evaluate their application in neurodegenerative diseases.

## 4. Materials and Methods

### 4.1. Reagents and Solutions

Tween^®^ 20 (Polyethylene glycol sorbitan monolaurate) (Sigma-Aldrich, Merck KGaA, Darmstadt, Germany), protease inhibitor cocktails (Thermo Fisher Scientific, Waltham, MA, USA), IGEPAL^®^ CA-630 (Octylphenoxy poly(ethyleneoxy)ethanol) (Sigma-Aldrich), and CZC-25146 (LRRK2 inhibitors) (Calbiochem, Merck KGaA). The phosphate-buffered saline (PBS) solution was made using NaCl (137 mM), KCl (2.7 mM), Na_2_HPO_4_ (8.1 mM), and KH_2_PO_4_ (1.47 mM) from Sigma-Aldrich and then adjusted to pH 7.4. Dulbecco’s modified Eagle’s medium (DMEM/F12), foetal bovine serum (FBS), streptomycin/penicillin, Geneticin-G418, and hygromycin were purchased from Thermo Fisher Scientific.

### 4.2. Plasmid Constructions

Plasmids coding WT or mutants LRRK2 and human Sec8 were previously described in [25].

### 4.3. Cell Lines

The PC12-TET-ON (Takara Bio, San Jose, CA, USA) and PC12-TET-ON-G2019S [50] cell lines were cultivated in DMEM/F12 supplemented with 10% tetracycline-free FCS (Lonza, Basel, Switzerland) at 37 °C. HEK 293T (ATCC number CRL-3216) was grown in DMEM (Thermo Fisher Scientific), 10% foetal calf serum (FCS, Thermo Fisher Scientific) at 37 °C.

### 4.4. Adenovirus Transduction

Adenovirus for LRRK2 expression and the transduction protocol were previously described [14]. SH-SY5Y were plated at 40% of confluence and, the day after, were transduced by recombinant adenovirus for 1 h in a serum-free medium. After 1 h, the medium was replaced by a medium containing 1% serum. At the indicated time points, the cells were washed twice by cold PBS 1X and lysed in Laemmli buffer 1X.

### 4.5. Drosophila Lines

All fly stocks were maintained in our laboratory on a standard cornmeal medium at 25 °C. The Actin-GAL4 driver (stock number 4414) and UAS-Sec8 (stock number 9556) were from Bloomington Stock Center (Bloomington, IN, USA). UAS-LRRK2 WT and UAS-LRRK2-R1441C were a generous gift from Prof. Cheng-Ting Chien (National Taiwan University Hospital Yun-Lin Branch, Taipei, Taiwan) [51].

### 4.6. Evaluation by RT-PCR of dSec8 mRNA Level in Drosophila Transgenic Lines

Total RNA was purified from three drosophila for each genotype in 500 µL of TRIZOL solution according to the manufacturer’s instruction (Thermo Fisher Scientific). An amount of 1 µg of total RNA was converted to cDNA by AMV reverse transcriptase (Promega, Madison, WI, USA) at 37°C for 1 h. PCR amplification was performed at 94 °C 30 s, 55 °C 30 s, and 72 °C 30 s (dSec8 forward AGCTCCGGAAGATGTGGATGG, reverse GAAGAGAGGGCCTCGTTGGC, L32 forward GACGCTTCAAGGGACAGTATCTG, reverse AAACGCGGTTCTGCATGAG).

### 4.7. Immunoprecipitation

HEK293 cells were transfected with the indicated plasmids in 35 mm cell culture plates by LTX/PLUS kit (Thermo Fisher Scientific). At the end of transfection (4 h), the cells were treated or not for 48 h by Edosidin2 (15 µM or 5 µM). The cells were then washed twice in PBS 1X and lysed by 1 mL of NP40 lysis buffer (150 mM NaCl, 1% NP40, 20 mM Tris-HCl pH 7.5, protease inhibitor cocktail). Cellular debris was removed by centrifugation at 13,000× *g* and cell lysates were pre-cleared by incubation with protein G-agarose beads for 1 h at 4 °C. The protein extracts were then incubated by anti-Flag antibody (F3165, 1:1000, Sigma-Aldrich) or anti-Myc (M4439, 1:1000, Sigma-Aldrich) overnight at 4 °C. After incubation with protein G-agarose for 1 h at 4 °C, the beads were washed four times by lysis buffer. Samples were then eluted in Laemmli buffer and resolved by SDS-PAGE.

### 4.8. Western Blot Analysis

Western blot analysis was performed as previously described [14]. In detail, protein extracts were prepared by direct lysis in Laemmli buffer or NP40 1% buffer when protein content was determined using the Bradford protein assay. Equal amounts of protein extracts were resolved by standard SDS-PAGE and subsequently electroblotted into nitrocellulose membrane (Thermo Fisher Scientific). The nitrocellulose membranes were incubated with 3% low-fat milk in 1X PBS-Tween 0.05% solution with the indicated antibody: anti-LRRK2 (1:5000 MJFF2 c41-2 Abcam, Cambridge, UK), anti-Flag (1:2500 F3165 Sigma-Aldrich), anti-Myc (M4439, 1:5000, Sigma-Aldrich), anti-beta-actin (A5441 1:5000 Sigma-Aldrich), anti-Sec8 (1:1000 610,659 BD Biosciences, San Jose, CA, USA), anti-Exo70 (1:1000 HPA022840 Sigma-Aldrich), and anti-α-tubulin (1:500 12G10 DSHB), for 16 h at 4 °C. Goat anti-mouse immunoglobulin G (IgG) peroxidase-conjugated antibody (1:2500 Millipore Corporation, Merck KGaA) or goat anti-rabbit IgG peroxidase-conjugated antibody (1:5000 Millipore Corporation) were used to identify the immunocomplexes by enhanced chemiluminescence (ECL start Euroclone SpA, Milano, Italy).

### 4.9. PC12-LRRK2 G2019S Differentiation and Analysis

PC12 cells stably expressing doxycycline (dox)-inducible LRRK2 G2019S mutant [50] were grown in DMEM/F12, 10% tet-free FBS (tetracycline-free FBS, Lonza) at 37 °C. For the differentiation experiment, the cells were plated at low density (5% of confluence) on a cover glass. The differentiation was performed growing the cells in 1% FBS in the presence of 100 ng/mL of NGF and doxycycline (0.2 μM) for 7 consecutive days, in the absence or presence of the different compounds: Endosidin2 (2.5 or 5 μM), CZC-25126 (1 μM), and levetiracetam (10 μM). The medium containing the different compounds was replaced every 48 h. At the end of the experimental procedure, the differentiated cells were fixed with 4% paraformaldehyde/PBS and analysed by immunofluorescence.

### 4.10. Immunofluorescence

The cells were plated and grown on a cover glass for the indicated time, washed twice with PBS 1X, and then fixed with 4% paraformaldehyde/PBS1X for 10 min. Cells were permeabilised with 0.1% Triton X-100 diluted in PBS1X. Non-specific binding was blocked with 5% bovine serum albumin (BSA), 0.05% Tween 20 diluted in PBS1X for 1 h at room temperature. Fixed cells were incubated with primary antibodies: anti-LRRK2 (1:500 MJFF2 c41-2 Abcam) and anti-Flag (F3165 1:2000 Sigma-Aldrich), diluted in blocking solution, overnight at 4 °C. Cells were then washed with PBS1X, 0.05% Tween 20 and incubated with secondary antibodies: Goat anti-Mouse IgG Secondary Antibody Alexa Fluor^®^ 488 (Thermo Fisher Scientific) and Goat anti-Mouse IgG Secondary Antibody Alexa Fluor^®^ 647 (Thermo Fisher Scientific), diluted 1:1000 in blocking solution for 1 h at room temperature. Finally, after adding the Mowiol mounting medium, the cells were analysed by Leica TCS SP5 confocal microscope with LAS lite 170 image software (Advance Fluorescence 2.7.3.9723).

### 4.11. Whole-Mount Immunostaining of the Adult Drosophila Brains

Fluorescent immunostaining was performed on whole-mount dissected adult brain at 45 days of age. Cohorts of 6 to 10 flies per genotype were used each time. Brains were fixed using 4% paraformaldehyde in phosphate-buffered saline (PBS), pH 7.4. After fixation, brains were permeabilised with 0.3% Triton X-100 in PBS for 20 min at room temperature (RT) and then incubated in blocking buffer (5% normal goat serum in PBS 1X-0.3% Triton X-100) for 1 h at RT. Subsequently, the incubation with primary antibody anti-TH (AB152 Millipore) diluted in blocking buffer was carried out for 48 h at 4 °C. After extensive washing, the brains were incubated by secondary antibody Alexa Fluor^®^ 488 (Thermo Fisher Scientific) diluted 1:1000 in blocking solution for 48 h at 4 °C. Before analysis, brains were mounted using the Mowiol mounting medium and fluorescence was revealed with a Leica TCS SP5 confocal microscope with LAS lite 170 image software. Statistical analysis was performed by two-way ANOVA followed by the appropriate post hoc test.

### 4.12. Climbing Assay

Male and female flies were age- and sex-matched, randomly selected, anaesthetised with ice, and placed in conical tubes with a diameter of 2 cm. After 15 min of recovery, the flies were tapped to the bottom of the tube, and their subsequent climbing activity quantified as the percentage of flies that reached 8 cm in 10 s. Any experimental sample was performed in duplicate (each with 15 flies) and the assay was repeated three times.

### 4.13. Statistical Analysis

The results are presented as means ± SEM of independent experiments as indicated. For bands analysis in Western blot experiments, after image acquisition, the protein bands were quantified by densitometry and normalised to the specific loading control using Quantity One software (Version 4.6.8, Biorad, Hercules, CA, USA). Statistical evaluation was conducted by the Student’s *t*-test or by one-way ANOVA and Bonferroni’s multiple comparison post-test. Values significantly different from the relative control are indicated with *, **, or *** symbols when *p* < 0.05, *p* < 0.01, and *p* < 0.001, respectively.

## Data Availability

The data presented in this study are available on request from the corresponding author.

## Supplementary Materials

The following supporting information can be downloaded at https://www.mdpi.com/article/10.3390/ijms241612656/s1.
