# Supplementary material for: Inhibition of the Exocyst Complex Attenuates the LRRK2 Pathological Effects
Source: Int J Mol Sci. 2023 Aug 10;24(16):12656. doi: 10.3390/ijms241612656 (PMC10454163; doi:10.3390/ijms241612656)
Supplement: Supplementary file 1 [file ijms-24-12656-s001.zip › ijms-2470406-supplementary.pdf]

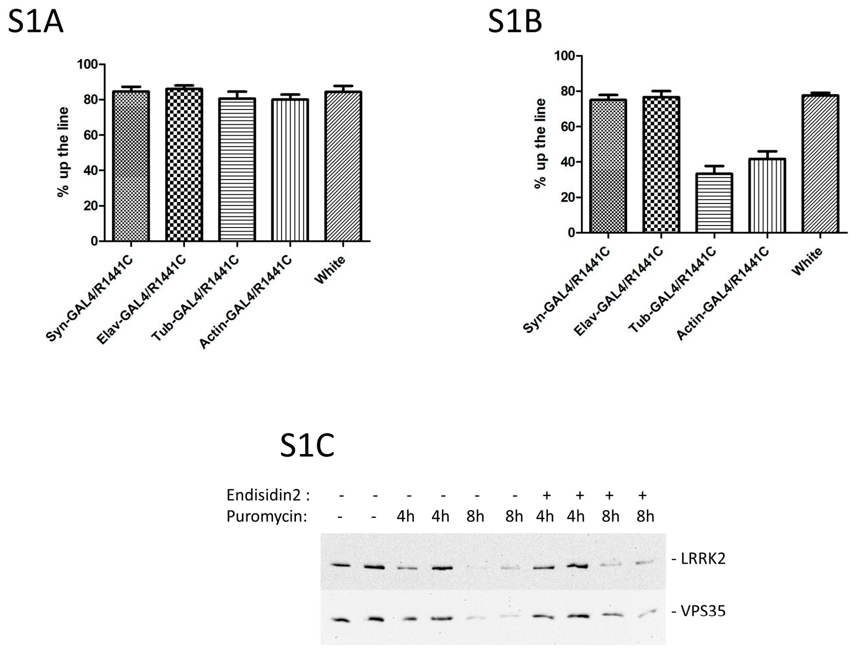

Figure S1. Evaluation of drosophila locomotor activity of LRRK2 transgenic animals under the control of different drivers and analysis of LRRK2 protein level upon Ensosidin2 treatment in SH-SY5Y cells. (**A-B**) Evaluation by climbing assay of locomotor activity of 7 days (A) or 30 days (B) old drosophila lines expressing LRRK2 R1441C under the control of Elav-GAL4, Syb-GAL4,Actin-GAL4 or Tub-Gal4 drivers. W* drosophila line was uses as control. (**C**) Analysis of LRRK2 degradation rate in the presence or absence of endosidin2. The SH-SY5Y cells were transduced as previously described in figure 4F and treated by puromycin for 4 or 8 hours in the absence or in the presence of endosidin2 (5µM). Western blot was performed by an anti-LRRK2 antibody and anti VPS35 was used as loading control
